# 3D Road Defect Mapping via Differentiable Neural Rendering and Multi-Frame Semantic Fusion in Bird’s-Eye-View Space

**DOI:** 10.3390/jimaging12020083

**Published:** 2026-02-15

**Authors:** Hongjia Xing, Feng Yang

**Affiliations:** School of Artificial Intelligence, China University of Mining and Technology (Beijing), Beijing 100083, China; yangf@cumtb.edu.cn

**Keywords:** neural reconstruction, multi-view geometry, 3D reconstruction, intelligent 3D vision, road defect detection, differentiable rendering

## Abstract

Road defect detection is essential for traffic safety and infrastructure maintenance. Excising automated methods based on 2D image analysis lack spatial context and cannot provide accurate 3D localization required for maintenance planning. We propose a novel framework for road defect mapping from monocular video sequences by integrating differentiable Bird’s-Eye-View (BEV) mesh representation, semantic filtering, and multi-frame temporal fusion. Our differentiable mesh-based BEV representation enables efficient scene reconstruction from sparse observations through MLP-based optimization. The semantic filtering strategy leverages road surface segmentation to eliminate off-road false positives, reducing detection errors by 33.7%. Multi-frame fusion with ray-casting projection and exponential moving average update accumulates defect observations across frames while maintaining 3D geometric consistency. Experimental results demonstrate that our framework produces geometrically consistent BEV defect maps with superior accuracy compared to single-frame 2D methods, effectively handling occlusions, motion blur, and varying illumination conditions.

## 1. Introduction

Road infrastructure maintenance is critical for ensuring traffic safety and economic efficiency. Pavement defects, including cracks, potholes, and alligator cracking, pose significant threats to vehicle safety and contribute to increased accident rates [1,2]. Traditional manual inspection methods are labor-intensive, time-consuming, and costly, limiting their effectiveness for large-scale road network monitoring [3]. With the rapid advancement of deep learning and computer vision technologies, automated road defect detection systems have emerged as promising solutions to address these challenges [4,5].

Recent progress in deep learning-based road defect detection has primarily focused on 2D image analysis using convolutional neural networks (CNNs) and object detection frameworks [6,7]. YOLO-based methods have demonstrated remarkable performance in real-time defect detection, with variants such as the improved YOLOv5 [8], YOLOv8 [9,10], and attention-enhanced architectures [11,12] achieving high accuracy on benchmark datasets like RDD2022 and GRDDC. However, these 2D detection approaches suffer from several limitations: (1) they lack spatial context and cannot provide accurate defect localization in 3D space, (2) single-frame detection is susceptible to occlusions, motion blur, and varying illumination conditions, and (3) they fail to leverage temporal consistency across video sequences for robust detection [13,14].

To address the spatial representation challenge, Bird’s-Eye-View (BEV) perception has gained significant attention in autonomous driving applications [15,16]. BEV representations transform multi-view camera observations into a unified top-down coordinate system, enabling intuitive spatial reasoning. Recent works such as BEVFormer [17], BEVFusion [18], and BEVerse [19] have demonstrated the effectiveness of BEV representations for 3D object detection and semantic segmentation. Li et al. [20] provide a comprehensive review of BEV perception, analyzing key challenges in view transformation and depth estimation, while Fast-BEV [21] proposes an efficient baseline achieving real-time performance. However, existing BEV methods primarily focus on dynamic object detection and have not been extensively applied to static infrastructure defect mapping.

Neural radiance fields (NeRF) [22] and differentiable rendering techniques [23,24] offer other promising directions for 3D scene reconstruction from 2D images. NeRF represents scenes as continuous volumetric functions using multi-layer perceptrons (MLPs), enabling photorealistic novel view synthesis [25,26]. Recent extensions have improved efficiency through advanced sampling strategies and hybrid representations [27,28,29]. Differentiable rendering enables gradient-based optimization of scene parameters directly from image observations [30,31]. For road infrastructure monitoring, specialized datasets [32] and detection methods have been developed. Aerial-based approaches such as AGSAM-Net [33] leverage UAV platforms for bridge inspection, while expert systems [34] incorporate domain knowledge for pothole assessment. However, these methods focus primarily on 2D detection or require specialized equipment, whereas our approach integrates video-based 3D reconstruction with temporal fusion.

Multi-frame temporal fusion has proven effective for enhancing detection robustness in video sequences [35,36]. Temporal aggregation methods leverage redundancy and consistency across frames to suppress noise, handle occlusions, and improve overall detection confidence. Recent approaches in video object detection employ deformable attention mechanisms, temporal context modeling, and adaptive frame selection strategies to effectively fuse information across time [37,38,39]. However, most existing multi-frame fusion methods operate in 2D image space and do not explicitly model 3D spatial structure, limiting their ability to produce geometrically consistent results for infrastructure mapping applications.

Despite these advances, several challenges remain in applying these techniques to road defect mapping: (1) 2D detection methods cannot provide accurate 3D localization required for maintenance planning, (2) BEV methods designed for autonomous driving focus on dynamic objects rather than static infrastructure, (3) NeRF-based reconstruction methods are computationally expensive and not optimized for large-scale outdoor scenes, and (4) existing multi-frame fusion approaches lack geometric consistency and produce 2D rather than 3D defect maps.

In this study, we propose a novel framework that addresses these limitations by integrating differentiable BEV mesh representation, semantic filtering, and multi-frame temporal fusion for accurate and robust road defect mapping from monocular video sequences. We focus on vision-based input using standard vehicle cameras due to its practical advantages: low deployment cost compared to LiDAR systems, easy integration with existing vehicle fleets, and scalability for road network monitoring. Our framework supports both monocular and multi-camera configurations, as demonstrated on KITTI (single front camera) and nuScenes (six-camera surround view) datasets. Our key contributions are threefold:

(1) Differentiable Mesh-based BEV Representation: We introduce an explicit mesh representation that combines the spatial reasoning advantages of BEV with geometric fidelity through multi-frequency positional encoding and MLP-based height prediction. Unlike implicit NeRF representations, our approach significantly reduces parameter complexity while enabling efficient scene reconstruction.
(2) Semantic-guided False Positive Filtering: We propose a filtering strategy that leverages road surface segmentation renders to eliminate off-road false positives. This mechanism operates on spatial overlap rather than classification confidence, reducing false-positive rates by 33.7% without sacrificing recall.
(3) Multi-frame Temporal Fusion with Geometric Consistency: We design a ray-casting projection mechanism combined with exponential moving average (EMA) update that maps 2D detections to 3D mesh vertices and accumulates observations across frames, naturally suppressing single-frame noise while maintaining 3D spatial consistency.

## 2. Problem Formulation and Framework Overview

### 2.1. Problem Definition

Given a sequence of onboard camera-captured road images ${\left\{I_{t}\right\}}_{t=1}^{T}$ along with corresponding camera parameters $\left\{K_{t},\left[R_{t}|t_{t}\right]\right\}$ and semantic labels $\left\{S_{t}\right\}$, our objective is to reconstruct the 3D geometric structure of the road surface while simultaneously localizing pavement defects in Bird’s-Eye-View (BEV) space. Formally, we aim to learn a mapping function:

(1) $$F:{\left\{I_{t},K_{t},\left[R_{t}|t_{t}\right],S_{t}\right\}}_{t=1}^{T}\to \left\{M_{geo},M_{def}\right\}$$

where $M_{geo}$ represents the 3D geometric model of the road surface, containing vertex positions, appearance, and semantic information, while $M_{def}(x,y)$ is a defect map in BEV space that encodes defect locations, types, and severity levels.

The input data for frame t consists of an RGB image $I_{t}\in R^{H\times W\times 3}$ capturing the visual appearance of the road scene, camera intrinsics $K_{t}\in R^{3\times 3}$ defining the projection from 3D camera coordinates to 2D image plane, camera extrinsics $\left[R_{t}|t_{t}]\in SE(3\right)$ (the Special Euclidean group of rigid transformations) representing rotation and translation in the world coordinate system, and semantic labels $S_{t}\in {\left\{0,1,\ldots ,C-1\right\}}^{H\times W}$ providing pixel-wise annotations including road-related categories such as lane markings, road surface, and curbs. The output geometric model comprises a vertex set $V={\left\{v_{i}\right\}}_{i=1}^{N}$ and their connectivity relationships, forming a structured mesh representation. The defect map $M_{def}$ is a scalar field defined on BEV plane coordinates (x,y), with values in [0,1] indicating the probability of defect presence at each spatial location.

This task presents three core technical challenges. First, how can we simultaneously optimize geometric structure, visual appearance, semantic labels, and defect information within a unified representation space? Traditional approaches typically address these aspects separately, where geometric reconstruction methods focus on depth and surface normals, while defect detection systems operate purely in 2D image space. Second, how can we accurately map defects detected in 2D images to 3D mesh vertices? This challenge involves establishing geometric correspondence between 2D detection bounding boxes and 3D spatial locations, as well as handling projection ambiguity when multiple vertices project to similar image regions and maintaining mapping accuracy under varying camera viewpoints and distances. Third, how can we leverage multi-frame observations to suppress single-frame detection noise while ensuring spatio-temporal consistency in defect localization? Single-frame detections are prone to false positives from shadows, lighting variations, and occlusions. Multi-frame fusion must effectively aggregate observations across time to enhance reliability without introducing artifacts from camera motion or scene dynamics.

For clarity in subsequent exposition, we define key mathematical notation used throughout this research. We denote $I_{t}$ for the RGB image at frame t; $S_{t}$ is the semantic segmentation map; $K_{t}$ and $\left[R_{t}|t_{t}\right]$ are the camera intrinsics and extrinsics respectively; V is the mesh vertex set with attributes; $V_{xy,i},V_{z,i},V_{rgb,i},V_{sem,i}$ are the fixed BEV coordinates, learnable height, appearance color, and semantic logits of vertex; $i,V_{obs,i},V_{sev,i},V_{conf,i}$ are the defect observation counts, accumulated severity, and maximum confidence; $D_{t}$ is the detection results at frame t consisting of bounding boxes $b_{x}$, class labels $c_{k}$, and confidence scores $conf_{k}$; and $M_{def}$ is the final BEV defect probability map.

We define geometric consistency as the property that defect locations mapped from different frames converge to the same 3D mesh vertices within a spatial tolerance of 2.5×r (where r is the mesh resolution). Formally, for a defect observed in frames $t_{1}$ and $t_{2}$, the mapped vertex positions $v_{1}$ and $v_{2}$ satisfy $\left\|v_{1}-v_{2}\right\|<2.5r$. This ensures that multi-frame observations reinforce rather than contradict each other in 3D space.

### 2.2. Overall Framework

**Figure 1:**
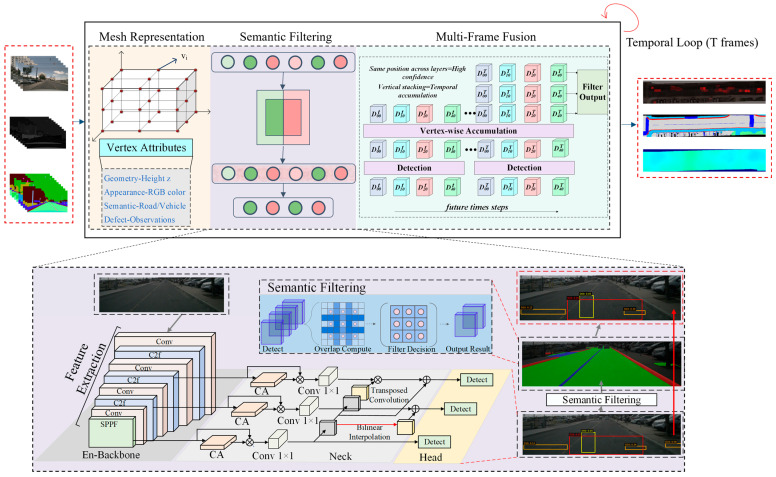
The overall framework of the proposed method. The system consists of three main components: (1) differentiable mesh representation with multi-layer perceptron (MLP) optimization for Bird’s-Eye-View (BEV) scene reconstruction, (2) semantic filtering to eliminate off-road false positives, and (3) multi-frame fusion through ray-casting projection and temporal accumulation. The bottom panel shows the detection network architecture with En-Backbone for feature extraction. Arrows indicate data flow and processing sequence. Color coding: red boxes represent input (left) and output (right), black boxes indicate intermediate processing modules, and blue boxes show the detection network architecture.

Our framework adopts a three-stage pipeline that progressively transforms 2D image observations into a comprehensive 3D BEV defect map. The architecture maximizes the synergy between geometric reconstruction and defect detection while maintaining computational efficiency through explicit mesh representation. Figure 1 presents the overall architecture of our method, which integrates differentiable mesh representation, semantic filtering, and multi-frame fusion to generate accurate BEV defect maps from monocular video sequences.

The first stage establishes a differentiable mesh representation (Section 3.1) that serves as the geometric foundation. We represent the road surface as a structured 2D grid in BEV space, where vertices are arranged regularly with fixed (x,y) coordinates and learnable height z. Each vertex encodes geometric attributes through height predicted via multi-frequency positional encoding and MLP, appearance attributes via RGB color optimized through photometric supervision, semantic attributes via class logits trained with semantic segmentation loss, and defect attributes including observation counts, severity, and confidence accumulated through multi-frame fusion. Through differentiable rendering, the mesh is projected into image space and jointly optimized via multi-task losses including RGB reconstruction, semantic segmentation, depth supervision, and geometric smoothness.

The second stage introduces semantic filtering for 2D defect detection (Section 3.2). While YOLOv8 detects defects in 2D images, raw detections contain numerous false positives on non-road regions, such as vehicles, pedestrians, and buildings. We leverage the use of learned semantic segmentation masks to compute the overlap ratio $R_{\mathrm{road}}$ between each detection box and road regions, retaining only detections with $R_{road}>\tau_{road}$. This filtering mechanism requires no additional training beyond existing semantic segmentation, operates efficiently through simple pixel statistics, provides strong geometric constraints based on spatial overlap, and experimental validation shows that it reduces false-positive rates by 33.8% on nuScenes without sacrificing recall.

The third stage achieves multi-frame fusion and 3D mapping (Section 3.3). Filtered 2D detections are mapped to 3D mesh vertices through ray-casting projection. For each detection, we back-project the detection box center into a 3D ray, find vertices within the distance threshold $d_{thresh}$ to the ray, accumulate observation counts $V_{obs}$ and update severity $V_{sev}$ via EMA, and update confidence $V_{conf}$ via maximum pooling. Multi-frame accumulation naturally suppresses single-frame noise as genuine defects receive repeated observations at consistent locations and amplifies their signal strength, while false detections fail to accumulate coherently. Finally, the BEV defect map is generated through spatial aggregation:

(2) $$M_{def}(x,y)=\sum_{v_{i}\in N(x,y)}G_{\sigma }\left(x-x_{i},y-y_{i}\right)\cdot P_{def}(v_{i})$$

where N(x,y) denotes the neighborhood vertex set around coordinates (x,y), $G_{\sigma }\left(\cdot \right)$ is a Gaussian kernel, and $P_{def}\left(v_{i}\right)$ is the defect probability computed from accumulated observations and severity.

**Figure 2:**
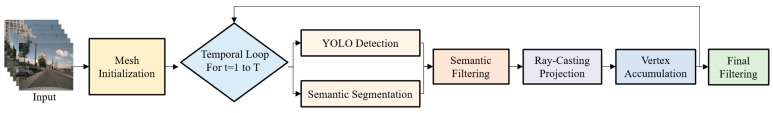
Processing pipeline. After mesh initialization, each frame undergoes YOLO detection and semantic segmentation, followed by semantic filtering to retain on-road detections. Ray-casting projects filtered 2D detections to 3D vertices, which are accumulated across T-frames. Final filtering by observation count produces high-confidence defect vertices.

The complete processing pipeline is illustrated in Figure 2, operating in a temporal loop over T-frames with iterative detection, filtering, projection, and accumulation.

This design addresses the three challenges identified above through complementary mechanisms. The unified mesh representation enables joint optimization of heterogeneous attributes while maintaining explicit topology for efficient BEV output. The ray-casting projection with adaptive thresholds establishes accurate 2D–3D correspondence robust to varying viewpoints and detection qualities. The EMA-based multi-frame fusion naturally enhances temporal consistency without requiring explicit motion models or feature tracking. Experimental results demonstrate that vertices associated with genuine defects receive an average of 2.48 observations on nuScenes, significantly boosting confidence compared to single-frame detections.

## 3. Methodology

This chapter presents the detailed methodology of our road defect detection and mapping framework. Figure 2 illustrates the overall processing pipeline. Building upon the problem formulation in Section 2, our framework operates in three sequential stages that progressively transform 2D image observations into a comprehensive 3D BEV defect map.

In the first stage, we construct a differentiable mesh-based BEV representation (Section 3.1) that serves as the geometric foundation. The road surface is modeled as a structured grid with fixed planar coordinates and learnable height values, optimized through multi-task losses including RGB reconstruction, semantic segmentation, and geometric smoothness. This stage produces both the 3D geometric model and the semantic segmentation masks required for subsequent filtering.

In the second stage, we perform semantic filtering for 2D defect detection (Section 3.2). While YOLOv8 detects potential defects in each frame, raw detections contain numerous false positives on non-road regions. We leverage the semantic segmentation masks from Stage 1 to compute road overlap ratios and filter out off-road detections, exploiting the principle that road defects must appear on actual road surfaces.

In the third stage, we achieve multi-frame fusion and BEV map generation (Section 3.3). Filtered 2D detections are mapped to 3D mesh vertices through ray-casting projection, and defect attributes are accumulated across frames using exponential moving average (EMA) update. This temporal aggregation naturally suppresses single-frame noise while maintaining geometric consistency, producing the final BEV defect map with localization, type, and severity information.

The three stages are tightly coupled, as Stage 1 provides both geometric structure and semantic priors for Stage 2, while Stage 2 supplies high-quality filtered detections for Stage 3’s temporal accumulation. This design maximizes the synergy between geometric reconstruction and defect detection.

### 3.1. Differentiable Mesh Representation

#### 3.1.1. Mesh Topology and Vertex Parameterization

Inspired by neural implicit representations, we design an explicit structured mesh representation that encodes multi-dimensional attributes of the road surface into vertex parameters. Compared to fully MLP-based implicit representations, explicit meshes maintain the efficiency of regular topology facilitating BEV output and defect mapping, while introducing local smoothness priors for geometric height through multi-frequency positional encoding and staged MLPs. This section describes the mesh topology and vertex parameterization, followed by the differentiable rendering process and multi-task joint optimization strategy.

Given the spatial extent $\left[0,L_{x}\right]\times \left[0,L_{y}\right]$ of the region of interest in BEV space, we perform regular sampling with a fixed resolution r (meters/cell) to generate mesh vertices. The number of vertices along the x and y directions are:

(3) $$n_{x}=\left\lceil \frac{L_{x}}{r}\right\rceil +1,n_{y}=\left\lceil \frac{L_{y}}{r}\right\rceil +1$$

yielding a total of $N=n_{x}\times n_{y}$ vertices. For KITTI $\left(L_{x}=L_{y}=600\;m,r=0.1\;m\right)$ and nuScenes $\left(L_{x}=L_{y}=100\;m,r=0.1\;m\right)$, the vertex counts are approximately $N_{KITTI}\approx 42.25\;M$ and $N_{KITTI}\approx 10.01\;M$, respectively. Considering dynamic cropping around the trajectory (cropping range 5–7 m), the actual number of used vertices is approximately $N_{eff}\approx 5k–15k$. The BEV plane coordinates of each vertex are fixed values:

(4) $$V_{xy,i}={\left[x_{\min}+(i\mathrm{mod}n_{x})\cdot r,\;y_{\min}+\left\lfloor \frac{i}{n_{x}}\right\rfloor \cdot r\right]}^{T}\in R^{2}$$

Adjacent vertices are connected through regular triangulation to form a set of triangular faces T, with approximately $2(n_{x}-1)(n_{y}-1)$ faces in total.

Each vertex $v_{i}$ is associated with multiple attribute categories. Geometric height $V_{z}\in R^{N_{eff}}$ is not directly optimized as $N_{eff}$ independent parameters, but is instead predicted through multi-frequency positional encoding and a two-stage MLP to ensure local smoothness. We first apply positional encoding to normalized vertex planar coordinates:

(5) $$\Phi (V_{xy,i})={\left[V_{xy,i},\;\sin(2^{0}\pi V_{xy,i}),\cos(2^{0}\pi V_{xy,i}),\ldots ,\sin(2^{L-1}\pi V_{xy,i}),\cos(2^{L-1}\pi V_{xy,i})\right]}^{T}$$

where L is the number of encoding layers (KITTI uses L=4, nuScenes uses L=5), and normalized coordinates $V_{xy,i}\in {\left[-1,1\right]}^{2}$. The encoded feature dimension is $d_{\Phi }=4L+2$ (18 for KITTI, 22 for nuScenes). Height values are predicted through a two-stage MLP. The first stage performs feature extraction:

(6) $$f_{0}={\mathrm{MLP}}_{0}(\Phi (V_{xy,i}))$$

where ${\mathrm{MLP}}_{0}$ consists of four linear layers (input dimension $d_{\Phi }$ → hidden dimension $d_{h}=128$ → $d_{h}$ → $d_{h}$ → $d_{h}$) with ReLU activation between layers. The second stage concatenates extracted features with original encoding for height prediction:

(7) $$V_{z,i}={\mathrm{MLP}}_{1}([\Phi (V_{xy,i});f_{0}])$$

where $\left[.;.\right]$ denotes feature concatenation, and ${\mathrm{MLP}}_{1}$ consists of four linear layers (input dimension $d_{\Phi }+d_{h}$ → $d_{h}$ → $d_{h}$ → $d_{h}$ → 1). This two-stage design enables height predictions of adjacent vertices to share smoothness constraints while reducing parameter count from $N_{eff}$ to approximately $d_{h}\left(d_{\Phi }+d_{h}+2\right)\approx 25k$, achieving a 99% reduction. MLP parameters $\Theta_{z}=\{W_{0},b_{0},\ldots ,W_{7},b_{7}\}$ are optimized through gradient descent during training, supporting full backpropagation.

Appearance color $V_{rgb}\in R^{N_{eff}\times 3}$ is directly optimized as a learnable parameter, initialized from a normal distribution $V_{rgb,i}\sim N(0,0.01I_{3})$. Semantic logits $V_{sem}\in R^{N_{eff}\times C}$ are similarly optimized as parameters, initialized to zero vectors $V_{sem,i}=0_{C}$ so that all vertices are uniform prior semantic label distributions (C=5 for KITTI, C=7 for nuScenes). Defect attributes including defect type $V_{type}\in R^{N_{eff}\times D}$ (one-hot encoding, D=4), severity $V_{sev}\in R^{N_{eff}}$ (scalar, range [0,1]), and observation confidence $V_{conf}\in R^{N_{eff}}$ (scalar) are not updated through gradients but passively accumulated through the ray-casting mechanism in Section 3.3. These attributes are initialized to zero and fuse multi-frame detection results through EMA.

The set of differentiable optimization parameters includes $\Theta =\Theta_{z}\cup \{V_{rgb,1},\ldots ,V_{rgb,N_{eff}}\}\cup \{V_{sem,1},\ldots ,V_{sem,N_{effs}}\}$, with the total parameter count approximately as:

(8) $$\left|\Theta |\approx d_{h}(d_{\Phi }+d_{h}+2)+N(3+C\right)$$

For KITTI ($N_{eff}\approx 8000,C=5$), this yields approximately 89.5 k parameters, while for nuScenes ($N_{\mathrm{eff}}\approx 15,000,C=7$) there are approximately 176 k parameters. This represents a 5–10× reduction compared to implicit NeRF’s million-scale parameters, and the explicit mesh topology also significantly accelerates training and inference.

**Figure 3:**
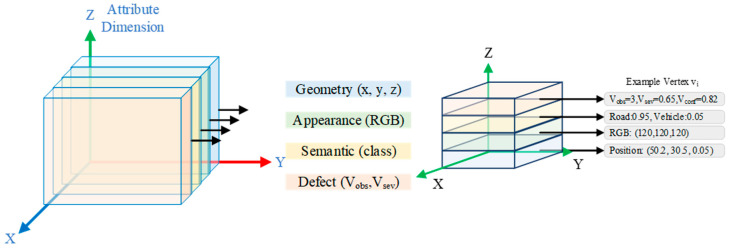
Multi-modal vertex representation. Each vertex stores four attribute types: (1) geometry (3D position and height); (2) appearance (RGB values); (3) semantic (class probabilities); and (4) defect (observation count $V_{obs}$, severity $V_{sev}$, confidence $V_{conf}$). Right panel shows an example vertex with typical attribute values.

Each vertex in the BEV mesh stores multi-modal attributes (Figure 3), enabling comprehensive representation of both geometric and defect information.

#### 3.1.2. Differentiable Rendering and Multi-Task Optimization

Given camera parameters $\left(K,\left[R|t\right]\right)$ and mesh vertex set V, we project vertices into image space through differentiable rasterization. The 3D coordinates of the i-th vertex are $P_{i}={\left[V_{xy,i},V_{z,i}\right]}^{⊤}\in R^{3}$. In camera coordinate systems, the coordinates become $P_{i}^{\mathrm{cam}}=RP_{i}+t$, and the homogeneous coordinates projected onto the image plane are:

(9) $$u_{i}=KP_{i}^{cam}=K(RP_{i}+t)$$

After removing homogeneous coordinates, pixel coordinates are ${\left[u_{i},v_{i}\right]}^{T}={\left[u_{i}[0]/u_{i}[2],u_{i}[1]/u_{i}[2]\right]}^{T}$ with depth ${\tilde{z}}_{i}=P_{i}^{\mathrm{cam}}[2]$. Through the rasterizer, for each pixel $p=\left(u,v\right)$, we determine its covering triangular face and barycentric coordinates. Assuming pixel p falls on triangle $\left(v_{i},v_{j},v_{k}\right)$, its barycentric coordinates $\left(\alpha_{p},\beta_{p},\gamma_{p}\right)$ satisfy:

(10) $$\left[\begin{array}{c} u\\ v \end{array}\right]=\alpha_{p}\left[\begin{array}{c} u_{i}\\ v_{i} \end{array}\right]+\beta_{p}\left[\begin{array}{c} u_{j}\\ v_{j} \end{array}\right]+\gamma_{p}\left[\begin{array}{c} u_{k}\\ v_{k} \end{array}\right],\;\alpha_{p}+\beta_{p}+\gamma_{p}=1$$

Barycentric coordinates are obtained by solving a 2 × 2 linear system, and this operation is fully differentiable with gradients back-propagatable to vertex projection coordinates. Rendered features at pixel p are obtained through barycentric interpolation of vertex attributes. For RGB channels, semantic logits, and depth, these are as follows:

(11) $$I_{render}(p)=\alpha_{p}V_{rgb,i}+\beta_{p}V_{rgb,j}+\gamma_{p}V_{rgb,k}$$

(12) $$S_{render}(p)=\alpha_{p}V_{sem,i}+\beta_{p}V_{sem,j}+\gamma_{p}V_{sem,k}$$

(13) $$D_{render}(p)=\alpha_{p}{\tilde{z}}_{i}+\beta_{p}{\tilde{z}}_{j}+\gamma_{p}{\tilde{z}}_{k}$$

To simultaneously optimize the mesh’s geometric, appearance, and semantic attributes, we employ four loss functions. RGB reconstruction loss measures pixel-wise differences using L1 norm for robustness: $L_{rgb}=\frac{1}{\sum_{p}M\left(p\right)}\sum_{p\in P}M\left(p\right)\cdot {\left\|I_{render}\left(p\right)-I_{gt}\left(p\right)\right\|}_{1}$, where $M\left(p\right)$ is a binary mask identifying valid pixels. Semantic segmentation loss supervises the rendered semantic map through cross-entropy: $L_{sem}=\frac{1}{\sum_{p}M\left(p\right)}\sum_{p\in P}M\left(p\right)\cdot CE\left(S_{render}\left(p\right)-S_{gt}\left(p\right)\right)$. For datasets with depth ground truth like KITTI’s LiDAR, depth supervision loss constrains mesh geometry: $L_{rgb}=\frac{1}{\sum_{p}M\left(p\right)}\sum_{p\in P}M\left(p\right)\cdot {\left\|D_{render}\left(p\right)-D_{gt}\left(p\right)\right\|}_{1}$, where $M_{d}\left(p\right)=M\left(p\right)\cdot 1\left[D_{gt}\left(p\right)>0\right]$ excludes invalid depth (for nuScenes without depth ground truth, we set $\lambda_{depth}=0$). Geometric smoothness regularization through Laplacian smoothness encourages neighboring vertices to have similar heights: $L_{smooth}=\frac{1}{\left|E\right|}\sum_{(i,j)\in E}{\left(V_{z,i}-V_{z,j}\right)}^{2}$, where ε is the set of mesh edges.

The four losses are combined into a total loss function:

(14) $$L_{total}=L_{rgb}+\lambda_{sem}L_{sem}+\lambda_{depth}L_{depth}+\lambda_{smooth}L_{smooth}$$

Using the Adam optimizer to update differentiable parameters: $\Theta^{\left(t+1\right)}=\Theta^{\left(t\right)}-\eta \cdot \mathrm{Adam}\left(\nabla_{\Theta }L_{\mathrm{total}}\right)$. To adapt to different optimization difficulties of attributes, we employ different learning rates for MLP parameters, RGB parameters, and semantic parameters (KITTI: $\eta_{rgb/sem}=0.1,\eta_{z}=0.001$; nuScenes: $\eta_{rgb/sem}=0.1,\eta_{z}=0.001$). After 7 epochs of training, the mesh gradually converges to a unified representation encoding geometry, appearance, and semantics, providing a geometric foundation for subsequent defect mapping.

**Figure 4:**
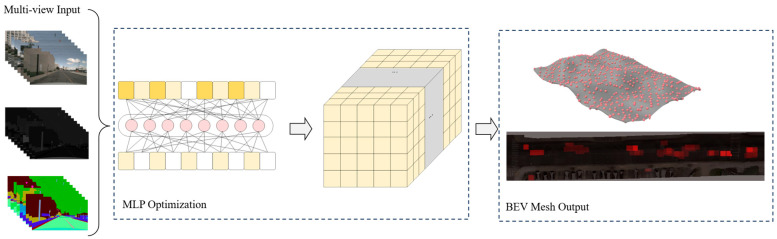
BEV mesh reconstruction via MLP optimization. Multi-view images are processed through an MLP network to optimize vertex positions, appearance (RGB), and semantic attributes. The network is trained using multi-task losses ($L_{rgb}$, $L_{sem}$, $L_{depth}$, $L_{smooth}$) to produce a continuous BEV mesh covering 100 × 100 m at 0.1 m resolution with 143,857 vertices. Colors in the layered structure represent different vertex attributes (geometry, appearance, semantics) optimized through MLP. Output shows the reconstructed BEV mesh with RGB and semantic information.

As illustrated in Figure 4, the MLP network learns vertex attributes from multi-view observations through differentiable rendering and multi-task optimization over seven epochs.

An important aspect of this multi-task optimization framework is the mutual enhancement between semantic segmentation and geometric reconstruction. Accurate geometric structure provides reliable spatial context for semantic predictions—for example, elevated curbs are more likely to be classified as “curb” rather than “road surface.” Conversely, semantic labels constrain the geometric optimization, such as regions labeled as “road surface” exhibit smooth, continuous height variations, while boundaries between different semantic classes may allow steeper height changes. This synergy is achieved through joint training rather than explicit coupling terms, and the learned road surface semantic segmentation will play a crucial role in Stage 2 (Section 3.2) by providing spatial constraints for defect detection filtering.

### 3.2. Semantic Filtering for 2D Defect Detection

**Figure 5:**
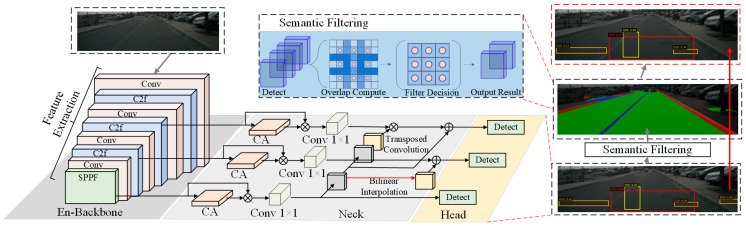
Semantic filtering process. The method computes the road overlap ratio $R_{road}$ for each detection using rendered semantic segmentation. Detections with $R_{road}\geq 0.5$ are retained while off-road detections are filtered out. The right panel shows results: (top) raw detections, (middle) the road mask, and (bottom) filtered on-road detections, reducing false positives by 33.7%.

YOLOv8 achieves strong detection performance on the RDD2022 dataset, but in complex road scenes it still produces numerous false positives in non-road regions, such as vehicles, pedestrians, and shadows. These false detections would accumulate spurious defect information in subsequent 3D mapping, degrading mesh quality. We leverage semantic segmentation masks to constrain 2D detection results, exploiting the principle that road defects must necessarily appear on actual road surfaces. This section describes the YOLOv8 detection output and the semantic segmentation-based road filtering mechanism. Figure 5 illustrates the semantic filtering process, which eliminates 33.7% of false positives by filtering detections based on the road overlap ratio.

We employ the YOLOv8 object detector pretrained on the RDD2022 dataset to identify four road defect types: longitudinal cracks (D00), transverse cracks (D10), alligator cracks (D20), and potholes (D40). For the input image sequence ${\left\{I_{t}\right\}}_{t=1}^{T}$, YOLOv8 outputs a set of detection results for each frame:

(15) $$D_{t}=\{(b_{k},c_{k},conf_{k})|\;k=1,\ldots ,K_{t}\}$$

where $b_{k}{\;=\;(x}_{1}{,\;y}_{1}{,\;x}_{2}{,\;y}_{2})$ is the bounding box coordinates of the k-th detection, $c_{k}\in \{0,1,2,3\}$ is the defect class label, and $conf_{k}\in [0,1]$ is the neural network output confidence score. YOLOv8 adopts an anchor-free detection paradigm with advantages in small target detection compared to traditional anchor-based methods. However, these raw detections present several issues, including confidence threshold settings affect detection recall; YOLOv8 is trained on RDD2022, which mainly contains isolated defect samples, and thus can easily and incorrectly detect objects in real road scenes, while viewpoint and illumination variations cause certain vehicle body parts or building facades to visually resemble defects. Relying solely on YOLOv8 confidence scores cannot sufficiently guarantee detection reliability.

To eliminate off-road false positives, we compute the overlap ratio between each detection box and road region:

(16) $$R_{road}(b_{k})=\frac{\left|\left\{p\in b_{k}:S_{t}(p)\in C_{road}\right\}\right|}{\left|b_{k}\right|}$$

where $C_{road}$ is the set of road-related semantic categories (specific values differ for KITTI and nuScenes, but all include primary road surface categories), and p denotes pixel location. The numerator counts pixels within the detection box that fall into the road categories, while the denominator $\left|b_{k}\right|$ is the total number of pixels in the box. The filtering rule only retains the detection boxes with an overlap ratio exceeding threshold $\tau_{raod}$:

(17) $$\mathrm{Accept}\;b_{k}\;\Leftrightarrow \;R_{\mathrm{road}}(b_{k})>\tau_{road}$$

Threshold selection balances overly low thresholds that retain too many off-road detections (high false positives) with overly high thresholds that excessively filter genuine defects near boundaries (high false negatives). Preliminary experiments on validation sets indicate that 0.5 is a reasonable balance point, requiring at least half of the pixels within detection boxes to fall in the road region.

This filtering approach differs from confidence-based methods in several aspects. Semantic filtering and detection confidence operate on orthogonal information sources—spatial overlap versus defect classification likelihood. Experimental validation in Section 4.5.2 demonstrates that the filter rate remains stable at approximately 34% across varying confidence thresholds (from 0.05 to 0.25), confirming the orthogonality between road/non-road discrimination and defect/non-defect confidence. The mechanism requires no additional training beyond existing semantic segmentation and operates efficiently through simple pixel statistics with time complexity $O\left(\left|b_{k}\right|\right)$ executable in batches on GPU, providing geometric constraints based on spatial overlap rather than learned patterns. Since non-road objects like vehicles typically do not belong to road regions, semantic segmentation effectively filters these out. Experimental results in Section 4.2 show that this mechanism reduces the false-positive rate by 33.8% on nuScenes.

Different defect types vary significantly in their impact on road safety and structural lifespan. We assign severity weights to each defect type based on international pavement distress rating standards: $w_{sev}\left(D00\right)=0.3$ for longitudinal cracks (most common but relatively minor); $w_{sev}\left(D10\right)=0.4$ for transverse cracks (moderate severity); $w_{sev}\left(D20\right)=0.7$ for alligator cracks (large-area damage with higher risk); and $w_{sev}\left(D40\right)=1.0$ for potholes (highest weight as they pose the most direct threat to driving safety). These weights are used in the multi-frame accumulation process in Section 3.3, where defect severity at vertices is updated via exponential moving average:

(18) $$V_{sev,i}\leftarrow \alpha \cdot w_{sev}(c_{k})+(1-\alpha )\cdot V_{sev,i}$$

where α is the EMA coefficient (set to 0.3) and $c_{k}$ is the current detection’s defect class. Through this mechanism, high-risk defects like potholes exhibit more pronounced accumulation effects. The weight settings can be adjusted according to specific maintenance policies and road classes, enabling the framework to adapt to different application scenarios.

Filtered detection results $D_{t^{\prime }}=\{(b_{k^{\prime }},c_{k^{\prime }},conf_{k^{\prime }})|(b_{k},c_{k},conf_{k})\in D_{t},R_{road}(b_{k})>\tau_{road}\}$ are passed to Section 3.3 for three-dimensional spatial mapping.

### 3.3. Multi-Frame Fusion and BEV Defect Map Generation

Single-frame 2D detection results are susceptible to illumination, occlusion, and detector uncertainty, leading to temporal instability. To obtain robust 3D defect localization, we sought to design a ray-mesh projection mechanism that maps 2D detection boxes to 3D mesh vertices, followed by multi-frame observation accumulation to enhance defect information reliability. The core principle aims to leverage camera extrinsics and intrinsics to establish 2D–3D correspondence, thereby accumulating multiple observations at mesh vertices to naturally suppress single-frame noise. This section describes the ray-to-mesh projection process and the multi-frame accumulation strategy through exponential moving average (EMA).

#### 3.3.1. Ray-Casting Projection from 2D to 3D

**Figure 6:**
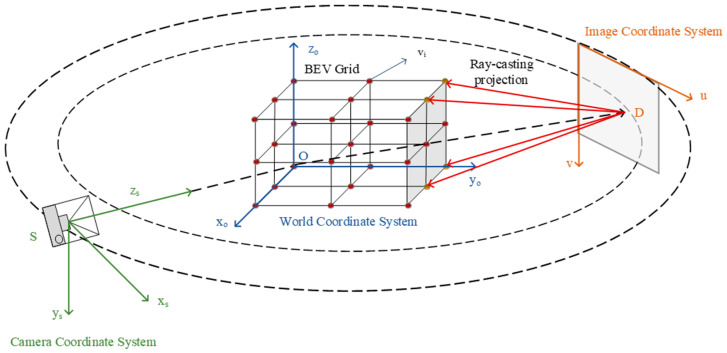
Coordinate systems and transformations. The ray-casting process involves: camera coordinate system (S), world coordinate system (O), image coordinate system (D), and BEV grid. Transformations are performed using camera intrinsic K and extrinsic $\left[R,t\right]$ parameters to project 2D detections to 3D vertices.

To establish 2D–3D correspondence, we perform coordinate transformations between multiple reference frames (Figure 6), enabling ray-casting from the image space to a BEV grid.

Given a filtered detection $(b_{k},c_{k},{\mathrm{conf}}_{k})\in {D_{t}}^{\prime }$ from frame t, we first compute the detection box center coordinates:

(19) $$(u_{c},v_{c})=\left(\frac{x_{1}+x_{2}}{2},\frac{y_{1}+y_{2}}{2}\right)$$

This center point represents the detection box’s position on the image plane. Through the inverse of the camera intrinsic matrix K, we back-project the image coordinates into a unit direction vector in the camera coordinate system:

(20) $$d_{\mathrm{cam}}=K^{-1}\left[\begin{array}{c} u_{c}\\ v_{c}\\ 1 \end{array}\right]$$

This vector’s direction points from the camera’s optical center toward the point $\left(u_{c},v_{c}\right)$ on the image plane. Combining camera extrinsics $\left[R_{t}|t_{t}\right]$ (rotation and translation), we obtain the ray’s parametric equation in world coordinates:

(21) $$r(\lambda )=t_{t}+\lambda \cdot R_{t}d_{\mathrm{cam}},\;\lambda \geq 0$$

where λ is the ray parameter representing distance from the camera position as $t_{t}$ along the ray direction.

For each mesh vertex $v_{i}={\left[V_{xy,i},V_{z,i}\right]}^{T}$, we compute its minimum distance to the ray. The point-to-ray distance is defined as:

(22) $$d_{i}=\min_{\lambda \geq 0}{\left\|v_{i}-r(\lambda )\right\|}_{2}$$

By solving $\frac{\partial }{\partial \lambda }{\left\|v_{i}-r(\lambda )\right\|}_{2}^{2}=0$, we obtain the optimal parameter:

(23) $$\lambda^{*}=\frac{{\left(v_{i}-t_{t}\right)}^{T}R_{t}d_{\mathrm{cam}}}{{\left(R_{t}d_{\mathrm{cam}}\right)}^{T}(R_{t}d_{\mathrm{cam}})}$$

and the corresponding minimum distance $d_{i}={\left\|v_{i}-r(\lambda^{*})\right\|}_{2}$. We establish a distance threshold as $d_{thresh}$ and associate vertices within this threshold with the detection:

(24) $$V_{\mathrm{assoc}}=\left\{v_{i}:d_{i}<d_{thresh}\right\}$$

**Figure 7:**
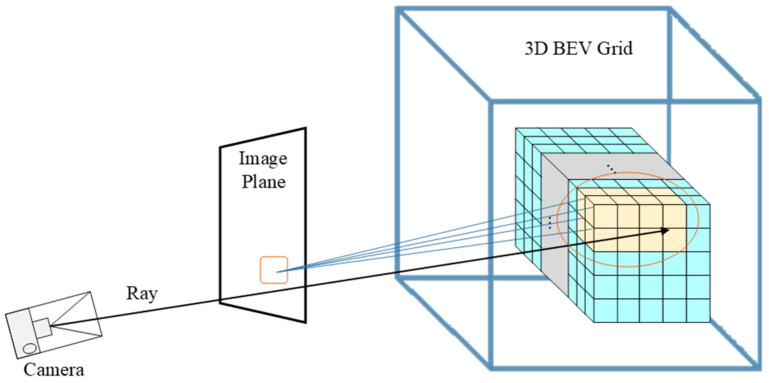
Ray-casting for 2D–3D association. A ray is cast from camera center through the detection center $u_{c},v_{c}$ into 3D space using $r\left(\lambda \right)=t+\lambda \cdot R\cdot K^{-1}\cdot \left[\begin{array}{ccc} u, & v, & 1 \end{array}\right]$. Vertices within the distance threshold $d_{thresh\;}=2.5r$ from the ray (highlighted region) are associated with the detection for attribute update.

The threshold setting considers several factors: mesh resolution r (finer resolution requires smaller threshold); camera distance (distant detections have greater uncertainty, allowing larger association range); and detection box size (larger boxes correspond to larger 2D projection areas, allowing more neighboring vertices to participate). In our implementation, we adopt an adaptive threshold:

(25) $$d_{thresh}=\beta_{0}\cdot r+\beta_{1}\cdot \left(1-conf_{k}\right)$$

where r is the mesh resolution, $conf_{k}$ is the detection confidence, and both $\beta_{0}$ and $\beta_{1}$ are tunable hyperparameters. High-confidence detections correspond to smaller thresholds (stricter association), while low-confidence detections allow larger association ranges (more tolerant fusion). This design ensures automatic adaptation to different detection qualities. For each filtered detection, ray-casting (Figure 7) identifies the associated BEV vertices within the distance threshold $d_{thresh}$ from the projected ray.

The ray-casting projection mechanism offers several technical advantages. It fully leverages camera extrinsic information to establish strict geometric correspondence, avoiding the need for explicit feature matching or depth prediction. The projection directly accumulates at mesh vertices without requiring intermediate 3D point cloud representation. The adaptive threshold ensures robustness as high-quality detections are precisely localized while low-quality detections are handled more permissively, preventing overly strict filtering and loose associations.

#### 3.3.2. Multi-Frame Observation Accumulation via EMA

Single ray-casting projection may contain errors due to camera calibration inaccuracy, detection box localization uncertainty, or mesh geometry imperfections. Through multi-frame observation fusion, we enhance defect localization stability. For each associated vertex $v_{i}\in V_{\mathrm{assoc}}$, we maintain defect class observation counts at mesh vertices:

(26) $$V_{obs,i}[c_{k}]\leftarrow V_{obs,i}[c_{k}]+1$$

where $V_{\mathrm{obs},i}[c_{k}]$ is the cumulative observation count for defect class $c_{k}$ at vertex $v_{i}$. This count vector has four components, represented as D=4, which correspond to four defect types. The observation count serves as a temporal confidence measure, where vertices receiving repeated observations across multiple frames indicate high-confidence defect presence, while vertices with only sporadic observations have likely resulted from false positives or noise.

Defect severity is not simply accumulated via counting but progressively updated through exponential moving average (EMA), giving greater weight to recent observations:

(27) $$V_{sev,i}\leftarrow \alpha \cdot w_{sev}(c_{k})+(1-\alpha )\cdot V_{sev,i}$$

where $w_{\mathrm{sev}}(c_{k})$ is the severity weight for the defect type in the k-th detection (weights defined as: D00 = 0.3, D10 = 0.4, D20 = 0.7, D40 = 1.0, based on international pavement distress standards), and $\alpha \in \left[0,1\right]$ is the EMA coefficient (set to 0.3 in this research). EMA provides three technical benefits: online updates without storing all historical observations, adaptive weighting where recent observations contribute more and can capture dynamic changes, and numerical stability that avoids overflow from simple averaging. The EMA coefficient α controls the response speed to new observations, where a large r α (e.g., 0.5) causes the model to react quickly but is susceptible to noise, while smaller α (e.g., 0.1) provides the opposite. Our setting of α=0.3 balances a rapid response to new observations (reaching 95% of new values after approximately 3–5 observations) with noise robustness.

Unlike the EMA update for severity, we adopt a maximum pooling strategy for detection confidence:

(28) $$V_{conf,i}\leftarrow \max(conf_{k},V_{conf,i})$$

**Figure 8:**
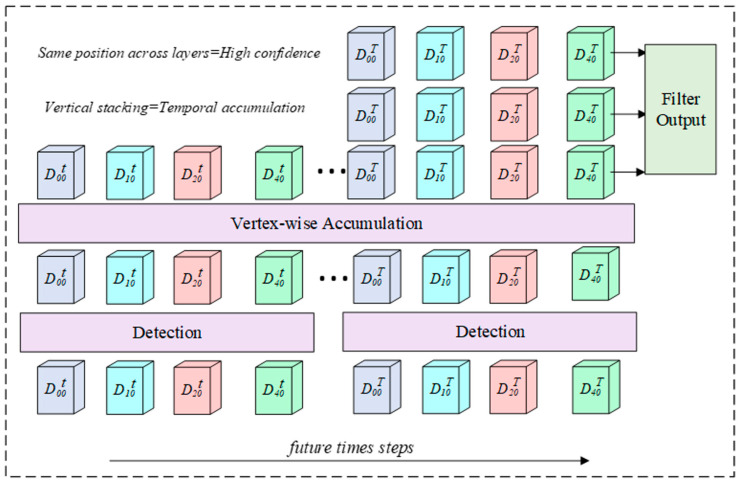
Vertex-wise temporal accumulation. Detections from multiple frames are projected to BEV vertices and accumulated over time. Vertices with consistent observations across frames (vertical alignment) exhibit high confidence. Observation count $V_{obs}$ and severity $V_{sev}$ are updated via EMA as $V_{sev}\leftarrow \alpha \cdot w_{sev}+\left(1-\alpha \right)\cdot V_{sev}$ with α=0.3.

The rationale is that confidence represents a single detector’s certainty that the sample belongs to a defect. If any observation across multiple frames has very high confidence, it sufficiently indicates the presence of a genuine defect at that location. Therefore, taking the maximum rather than an average is more reasonable. This design differs from traditional Kalman filtering or particle filtering approaches that explicitly model temporal dynamics—our method achieves similar noise suppression through passive accumulation without requiring motion models or feature tracking. The temporal accumulation process (Figure 8) maintains observation statistics for each vertex, with severity scores updated through exponential moving average across frames.

The multi-frame accumulation mechanism’s core advantage lies in natural suppression of single-frame detection noise. This can be considered through two scenarios: (1) Genuine defects where the same location’s defect is repeatedly detected across multiple adjacent frames. Through ray-casting projection, these detections accumulate at identical or neighboring mesh vertices, causing $V_{obs,i}$ and $V_{sev,i}$ to gradually increase and signal strength to amplify. (2) False-positive noise, where single-frame false detections (like vehicles or shadows) are rarely repeatedly detected at the same location across adjacent frames, meaning that these observations $V_{obs,i}$ do not significantly increase while keeping signal strength low. This natural signal-to-noise ratio improvement requires no explicit temporal models as it embodies design simplicity and effectiveness. Experimental results show that defect vertices on nuScenes receive an average of 2.48 observations, significantly boosting confidence (Section 4.3).

#### 3.3.3. BEV Defect Map Generation

After multi-frame accumulation, mesh vertices encode rich defect information. To generate the final BEV defect map $M_{\mathrm{def}}(x,y)$, we first compute each vertex’s defect existence probability:

(29) $$P_{def}(v_{i})=\sigma \left(V_{sev,i}\cdot \log(1+V_{obs,i}^{total})\right)$$

where $\sigma \left(\cdot \right)$ is the sigmoid function, $V_{obs,i}^{total}=\sum_{d=0}^{D-1}V_{obs,i}[d]$ is the total defect observation count at vertex i. This formula considers two factors, where severity $V_{\mathrm{sev},i}$ reflects average defect severity at that vertex, while observation strength $\log(1+V_{obs,i}^{total})$ uses a logarithmic function that grows quickly with few observations (emphasizing sparse observation importance) and slowly with many observations (avoiding over-emphasis on repeated observations). The product of both, transformed via sigmoid, maps to the [0, 1] range, representing defect existence probability.

Since vertex discretization may cause output map discontinuity, we perform Gaussian-weighted aggregation of neighboring vertices’ defect probabilities:

(30) $$M_{def}(x,y)=\sum_{v_{i}\in N(x,y)}G_{\sigma }(x-x_{i},y-y_{i})\cdot P_{def}(v_{i})$$

where N(x,y) is the neighborhood vertex set around coordinates $\left(x,y\right)$, and $G_{\sigma }(\cdot )$ is a Gaussian kernel with variance σ. This aggregation process produces smooth defect heatmaps facilitating subsequent risk assessment and visualization. For further refinement, we output defect maps decomposed by type. For each defect class c, its probability at vertex $v_{i}$ is defined as:

(31) $$P_{def}(v_{i},c)=\frac{V_{obs,i}[c]}{V_{obs,i}^{total}+\int }\cdot P_{def}(v_{i})$$

where ϵ is a small smoothing constant that avoids division by zero. This formula allocates total defect probability to each class proportionally by observation frequency. Through this decomposition, we generate specialized maps by defect type for targeted maintenance decisions.

The final BEV defect map $M_{\mathrm{def}}(x,y)$ and type-decomposed sub-maps support multiple applications: heatmap visualization for maintenance priority ranking (high-probability regions 0.7–1.0 require immediate repair, medium regions 0.4–0.7 need regular inspection, low regions 0.0–0.4 have no urgent needs); temporal tracking by saving maps from different epochs to monitor defect evolution trends at the same location; and integration with autonomous driving by incorporating defect maps into HD maps to provide road quality warnings for path planning and deceleration decisions.

## 4. Experiments and Results

This chapter evaluates the proposed fusion framework on two autonomous driving datasets. We first describe the experimental setup (Section 4.1), then validate fusion quality through semantic filtering effectiveness (Section 4.2), multi-frame fusion consistency (Section 4.3), and 3D mapping accuracy (Section 4.4). Ablation studies (Section 4.5) verify key design choices, qualitative results (Section 4.6) provide visual insights, cross-dataset robustness (Section 4.7) demonstrates the generalization capability, and limitations (Section 4.8) discuss the remaining challenges.

### 4.1. Experimental Setup

#### 4.1.1. Datasets and Scenarios

We evaluate the fusion framework on two autonomous driving datasets with diverse scene characteristics.

From the nuScenes dataset, we selected five representative scenes covering diverse environmental conditions and traffic patterns: Scene-0063 is an exit passage with trucks waiting at intersection; Scene-0064 shows parking lots with oncoming special electrical vehicles; Scene-0200 displays parking lots with parked cars; Scene-0655 features a complex parking lot with parked cars, jaywalkers, bendy buses, and gardening vehicles; and Scene-0283 captures a right-turn intersection with a policeman handling traffic.

For cross-dataset evaluation, we use KITTI Odometry sequence 00 [40] which contains 4541 frames covering a 3.7 km urban street trajectory. KITTI-00 features city roads with stable lighting and relatively open scenes, contrasting with nuScenes’ complex urban parking scenarios.

Table 1 summarizes the statistical characteristics of the selected scenes. All nuScenes scenes [41] contains 39–41 frames captured under daytime conditions but with varying traffic densities from sparse (Scene-0063) to dense (Scene-0200, 0655). Mesh vertex counts range from 131 K to 150 K vertices with 100 × 100 m spatial coverage, while KITTI-00’s longer trajectory uses 3.17 M vertices covering 600 × 600 m region. The YOLOv8 detector identifies varying numbers of potential defects across scenes, from 1452 total detections in KITTI-00 to 4908 in Scene-0064.

#### 4.1.2. Implementation Details

The fusion framework is implemented in PyTorch 1.10.2 with CUDA 11.3. Mesh representation uses BEV configuration with 600 × 600 m (KITTI) and 100 × 100 m (nuScenes) spatial regions, both at 0.1 m resolution. Geometric representation employs multi-frequency positional encoding (frequency = 4 for KITTI, frequency = 5 for nuScenes). Mesh height is refined through a two-stage MLP network optimized during training.

Training uses Adam optimizer with learning rates: $\eta_{rgb}=0.1,$ $\eta_{sem}=0.1,$ $\eta_{z}=0.001,$ $\eta_{pose}=0.001$. Loss weights are $\lambda_{sem}=0.5$ and $\lambda_{smooth}=1.0$. Training runs for seven epochs with 1000 frames per epoch on RTX 4080 GPU, requiring 45 min to 1 h per scene depending on server resource availability.

For defect detection, we use YOLOv8 pretrained on RDD2022. Detection confidence threshold is set to 0.05 (nuScenes) and 0.10 (KITTI) to maximize recall. Semantic filtering uses a road overlap threshold $\tau_{road}=0.5$. Ray-casting projection employs an adaptive distance threshold $d_{thresh}=2.5r$. Multi-frame fusion uses an EMA coefficient α=0.5. Implementation parameters are presented in Table 2.

#### 4.1.3. Evaluation Metrics

We evaluate fusion quality from three complementary perspectives.

Semantic filtering effectiveness is measured by the filter rate (FR) and on-road precision (ORP). The filter rate is the percentage of detections identified as off-road: $FR=\frac{N_{filtered}}{N_{total}}\times 100\%$. On-road precision is the complement: $ORP=\left(1-FR\right)\times 100\%$.

Multi-frame fusion quality is evaluated by coverage (Cov), observations per vertex (Obs/V), and average severity. Coverage is the percentage of vertices with defect observations: $Cov=\frac{N_{defect-vertices}}{N_{total-vertices}}\times 100\%$. Observations per vertex is the mean number of detections accumulated per defect vertex.

3D mapping accuracy is primarily assessed by mapping success rate (MSR), defined as the percentage of valid detections successfully projected to 3D mesh: $MSR=\frac{N_{mapped}}{N_{valid}}\times 100\%$. We also record average distance from mapped detections to their nearest vertices.

Our fusion framework achieves PSNR = 26.43 and 92.05% semantic segmentation accuracy on nuScenes. YOLOv8’s pretrained performance on RDD2022 is mAP@0.5 = 0.58.

### 4.2. Semantic Filtering Effectiveness

Table 3 presents the semantic filtering performance across different scenes. The filtering mechanism achieves an average filter rate of 33.8% on nuScenes with a standard deviation of only 4.1%, indicating stable performance across diverse scene conditions. This means approximately one-third of YOLOv8’s raw detections are successfully identified and removed as off-road false positives. On-road precision remains consistently around 60–73%, confirming that the retained detections are concentrated on actual road surfaces.

As shown in Table 3, filter rates correlate with scene complexity. Scene-0063, an exit passage with sparse traffic, exhibits the lowest rate due to fewer off-road objects. In contrast, Scene-0655, a dense parking environment with jaywalkers and vehicles, shows the highest rate. KITTI-00 achieves a 36.9% filter rate, slightly higher than the average for nuScenes, validating generalization across different datasets and camera configurations. The substantial proportion of filtered detections across all scenes demonstrates the practical necessity of semantic filtering. Without this step, approximately one-third of detections would be false positives originating from non-road objects such as parked vehicles and building facades. The method scales effectively to high-density detection scenarios while maintaining consistent filtering performance.

**Figure 9:**
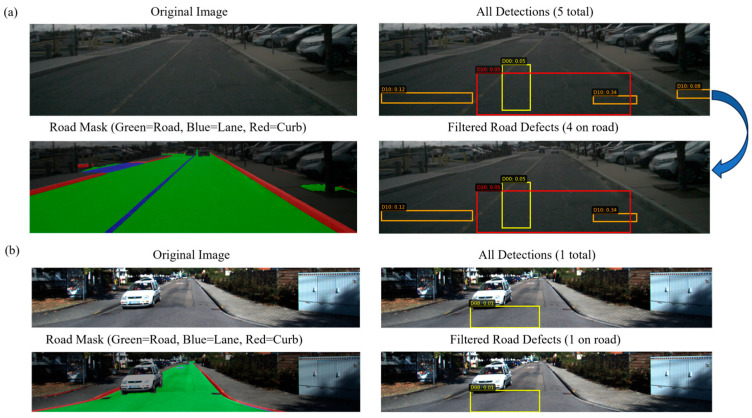
Visualization of semantic filtering process. (**a**) Scene-0064 shows raw YOLOv8 detections with off-road false positives on vehicles. (**b**) KITTI-00 demonstrates accurate road/non-road discrimination despite vehicle occlusion.

Figure 9 illustrates typical filtering examples. The upper panel shows Scene-0064 where YOLOv8 has detected five potential defects, including several as parked vehicles. Semantic filtering correctly removes one off-road detection while retaining four road-surface defects. The lower panel shows KITTI-00 where filtering accurately distinguishes the road surface from vehicles despite partial occlusion, demonstrating robust performance even under challenging conditions.

### 4.3. Multi-Frame Fusion Consistency

Table 4 summarizes the quality improvements from multi-frame fusion. The most critical metric is observations per vertex (Obs/V), reaching an average of 2.48 on nuScenes and 2.26 on KITTI-00, meaning each defect location is validated by multiple frames, significantly boosting confidence compared to single-frame detections.

Defect coverage varies meaningfully across scenes, reflecting actual road health differences rather than algorithmic inconsistency. Actively used parking areas exhibit higher coverage due to accumulated vehicle wear, while well-maintained exit passages show lower defect density. The consistent severity levels across both datasets indicate light-to-moderate damage typical of regularly maintained urban roads, validating that our severity weighting scheme produces comparable assessments across different road types and camera configurations.

**Figure 10:**
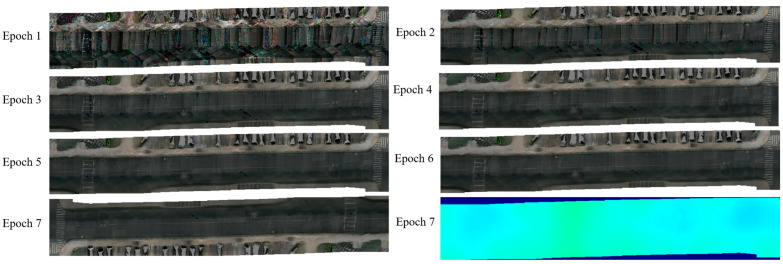
Evolution of multi-frame fusion process. The figure shows the temporal progression of BEV RGB reconstruction from Epoch 1 to Epoch 7, with progressively clearer lane markings, pavement texture, and vehicle details. The depth map (right) at Epoch 7 demonstrates improved geometric accuracy essential for precise 3D defect mapping.

Figure 10 visualizes the evolution in Scene-0064 across seven training epochs. The left series demonstrates progressive improvement in BEV RGB reconstruction quality. At Epoch 1, sparse texture and geometric irregularities reflect incomplete optimization. By Epoch 7, sharp lane markings, clear pavement texture, and accurate vehicle positions demonstrate converged reconstruction. The depth map comparison confirms the improved geometric stability that is crucial for accurate 3D defect mapping.

### 4.4. 3D Mapping Accuracy

Table 5 reports the mapping performance from 2D detections to 3D mesh vertices. KITTI-00 achieves higher mapping success compared to nuScenes, reflecting the impact of scene characteristics. KITTI’s regular city streets provide more favorable conditions for depth estimation than nuScenes’ cluttered parking scenarios with frequent occlusions and irregular geometry. This cross-dataset difference confirms that mapping performance depends critically on scene structure rather than detection quality alone.

For successfully mapped detections, spatial accuracy validates this conservative strategy. Average projection distances of 0.16 m (nuScenes) and 0.12 m (KITTI) remain well below the 0.25 m threshold, confirming sub-decimeter precision when depth information is valid. This accuracy level is sufficient for guiding repair crews, as typical road maintenance equipment operates at similar spatial tolerances. The fewer errors in KITTI further supports the observation that simpler scene geometry enables more precise geometric reasoning.

### 4.5. Ablation Studies

We conduct three ablation experiments targeting semantic filtering threshold, detection confidence threshold, and multi-frame fusion strategy. All experiments use Scene-0064 for controlled conditions.

#### 4.5.1. Road Filtering Threshold

Table 6 presents performance comparison under three different threshold settings. When $\tau_{road}$ varies from 0.3 to 0.7, the filter rate remains within 33.7–33.9%, coverage stays at 0.26–0.27%, and defect vertices range 373–388, demonstrating strong robustness. This stability stems from road segmentation quality characteristics where genuine road detections and clear off-road detections have distinct separation.

Based on the results in Table 6, we set $\tau_{road}=0.5$ as the default, aligning with widely used Intersection over Union (IoU) threshold 0.5 for intuitive interpretation while achieving balanced performance.

#### 4.5.2. Detection Confidence Threshold

Table 7 shows system performance under four different confidence settings. As confidence threshold increases from 0.05 to 0.25, the total detections drop 58%, directly causing coverage to decline 58%. Notably, the filter rate remains stable at ~33–35% despite varying confidence, revealing orthogonality between semantic filtering and detection confidence.

The stable filter rate across confidence thresholds in Table 7 confirms that semantic filtering operates on spatial overlap independent of classification confidence. We set $\tau_{conf}=0.05$ as the default to prioritize comprehensiveness, as the framework accumulates evidence across multiple frames to validate defects.

#### 4.5.3. Multi-Frame Fusion Strategy

Table 8 compares three different EMA weight settings. The EMA weight α varying from 0.5 to 0.9 has minimal impact on final metrics. These small variations demonstrate the method’s robustness to EMA weight selection within reasonable ranges. This insensitivity stems from the relatively short seven-epoch training period where historical information accumulation is limited. The slight increase in Obs/V and severity with higher α values suggests marginally better retention of historical observations, but the practical impact is minimal. Based on Table 8 results, we set α=0.5 as the default, prioritizing responsiveness to new detections while maintaining moderate historical context.

### 4.6. Qualitative Results

**Figure 11:**
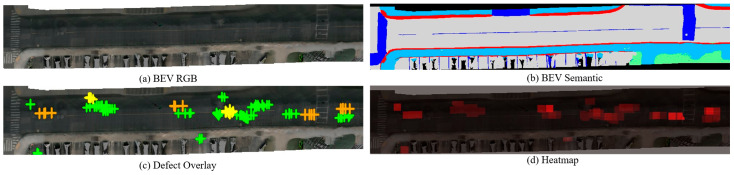
Final BEV defect map for Scene-0064. (**a**) BEV RGB reconstruction. (**b**) Semantic segmentation. (**c**) Defect overlay (green: D00 longitudinal cracks, yellow: D10 transverse cracks, orange: D20 alligator cracks, red: D40 potholes). (**d**) Statistical summary showing defect type distribution.

Qualitative visualization provides intuitive understanding of the fusion framework’s mechanisms and output quality. Figure 11 presents the final BEV defect map for Scene-0064 after the complete fusion pipeline, showing four complementary views. The high-fidelity RGB reconstruction captures road texture and lane markings, semantic segmentation distinguishes surface categories, and 375 defect vertices are accurately localized with type-specific annotations. The defect distribution predominantly features longitudinal cracks with moderate alligator cracking, reflecting typical urban parking lot deterioration patterns where repeated vehicle loading induces directional stress.

**Figure 12:**
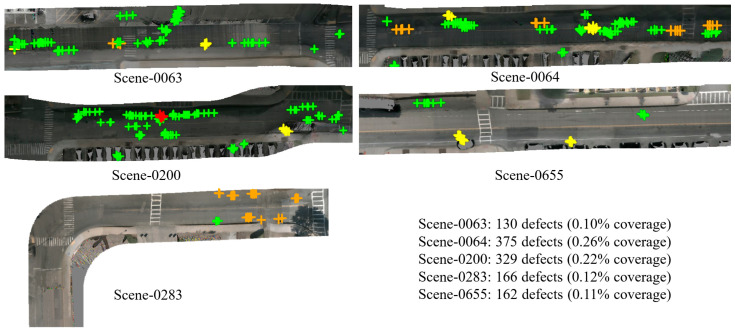
Cross-scene defect distribution comparing five representative nuScenes scenes. Color coding: green = D00 longitudinal cracks, yellow = D10 transverse cracks, orange = D20 alligator cracks, red = D40 potholes. Scene-specific statistics are listed on the right.

Figure 12 provides a comparative perspective across five representative scenes. Each scene’s BEV defect map reveals different defect patterns under varying road environments. Scene-0064 shows the highest coverage with defects distributed throughout the active parking area. Scene-0063 and Scene-0655 display lower coverage, indicating better maintained surfaces. Defect-type distribution varies meaningfully, as parking lot scenes show higher alligator crack proportions due to sustained vehicle pressure and aging.

**Figure 13:**
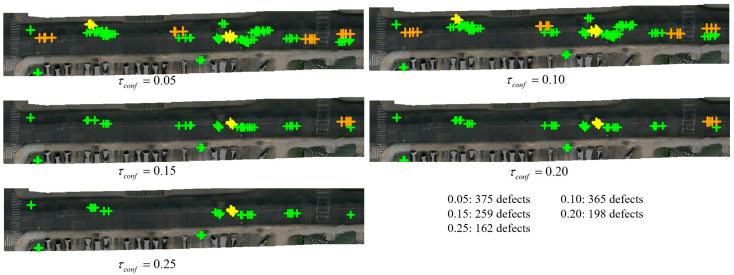
Confidence threshold ablation visualization for Scene-0064, showing progressive defect map refinement. Color coding: green = D00 longitudinal cracks, yellow = D10 transverse cracks, orange = D20 alligator cracks, red = D40 potholes.

Figure 13 demonstrates the impact of different detection confidence thresholds on Scene-0064’s BEV defect map. Progressive threshold increases from $\tau_{conf}=$ 0.05 to 0.25 reduces defect vertices from 375 to 162, quantifying the recall–precision tradeoff. At the loosest setting, the map captures all possible defects ensuring comprehensive coverage. At the strictest setting, retained defects have high confidence with stronger evidence. Even at the loosest setting, final maps maintain reasonable quality after semantic filtering and multi-frame fusion.

### 4.7. Cross-Dataset Robustness

Table 9 compares core performance metrics between nuScenes and KITTI datasets, revealing cross-dataset robustness. Despite significant differences between datasets—nuScenes uses six-camera surround view capturing 39–41 frames per scene in complex parking lots, while KITTI employs a single forward camera capturing 4541 frames along urban streets—core performance metrics maintain satisfactory consistency. The filter rate differs by only three percentage points, observations per vertex are comparable, and average severity shows agreement.

Coverage is the only metric in Table 9 that shows significant difference between the datasets, where nuScenes averages 0.16% while KITTI-00 approaches 0.00% (~0.004%). However, this reflects inherent dataset characteristics rather than method deficiency—KITTI-00 consists of well-maintained city streets with minimal visible distress, whereas nuScenes includes urban parking lots with naturally higher wear patterns. The extremely low but non-zero coverage in KITTI-00 confirms the method can detect sparse defects, even in well-maintained environments.

Within nuScenes, cross-scene performance shows reasonable variation with filter rate ranges of 27.5–39.6%, reflecting genuine scene complexity differences rather than algorithmic instability. The stable performance across different camera configurations and road geometries confirms the fusion framework’s strong generalization capability.

### 4.8. Limitations and Discussion

Despite strong overall performance, certain conditions reveal remaining limitations that merit discussion. We organize these limitations into five categories and discuss potential solutions for future work.

Lighting and Weather Conditions: System performance is highly contingent upon favorable visual conditions. While all evaluated scenes feature daytime lighting, challenging conditions such as evening/night scenes or adverse weather (rain, fog, snow) would cause detection performance to degrade significantly. This stems from YOLOv8’s inherent limitations under low illumination where defect visual features become blurred or indistinguishable from shadows. Additionally, wet road surfaces may cause specular reflections that interfere with both defect detection and semantic segmentation. Future work could address these limitations through domain adaptation techniques, low-light image enhancement preprocessing, or training detectors on diverse weather conditions.

Semantic Segmentation Reliability and Error Propagation: In cluttered urban environments with multiple dynamic objects and frequent occlusions, semantic segmentation quality may degrade, potentially leading to incorrect road/non-road classification. A critical concern is whether erroneous segmentation could propagate through the EMA-based accumulation process and corrupt mesh attributes. Our current design provides implicit safeguards: (1) the road overlap threshold $\tau_{road}=0.5$ requires majority agreement, making the filter robust to minor segmentation errors; (2) genuine defects receive repeated observations at consistent 3D locations across multiple frames, while false positives caused by segmentation errors tend to appear sporadically and fail to accumulate coherently; and (3) the observation count threshold in final filtering further suppresses low-confidence vertices. However, systematic segmentation failures in specific regions could still lead to persistent errors. Incorporating segmentation uncertainty estimation and confidence-weighted accumulation represents a promising direction for more robust error handling.

Depth Estimation and Geometric Mapping Accuracy: The mapping success rate of approximately 28% is constrained by the reliability of rendered depth. Depth estimation becomes unreliable at long distances (>50 m) and in heavily occluded regions where neural rendering lack sufficient multi-view constraints. Our current approach employs a simple adaptive threshold to filter unreliable depth values, but this strategy may be overly conservative, rejecting valid detections, or insufficiently strict, admitting erroneous mappings. A more rigorous approach would involve quantifying the error distribution of geometric mapping as a function of distance and view coverage. Future work could integrate learned monocular depth estimation networks (e.g., Depth Anything [42,43]) to provide more reliable depth priors, particularly for distant regions where rendered depth is unreliable.

Computational Efficiency and Scalability: Processing a sequence of approximately 40 frames requires 45 min to 1 h on an RTX 4080 GPU (16 GB VRAM), with the primary bottlenecks being differentiable mesh rendering and per-frame YOLOv8 inference. While acceptable for offline processing and research prototyping, this computational cost poses challenges for scaling to large urban road networks requiring thousands of sequences. The current framework does not provide a rapid inference mode that bypasses the per-scene optimization process. Potential optimization strategies include: (1) parallel processing of independent road segments, (2) lightweight backbone networks for semantic segmentation, (3) incremental mesh updates rather than full re-optimization, and (4) leveraging pre-trained geometric priors to reduce convergence iterations. We note that our primary contribution lies in demonstrating the feasibility of fusing geometric reconstruction with defect detection; computational optimization for real-time deployment remains an important direction for future engineering efforts.

Temporal Modeling and Consistency: The current framework adopts a “passive accumulation” strategy for consecutive frames without explicit modeling of temporal dynamics. This design choice offers simplicity and robustness—no motion models or feature tracking are required—but it has limitations. Transient observation gaps caused by occlusions or detector failures may lead to discontinuities in defect localization, as the system cannot actively predict defect presence in unobserved frames. For long video sequences in highly dynamic environments, the lack of temporal prediction mechanisms may limit processing efficiency. Future work could incorporate explicit temporal consistency constraints through techniques such as Kalman filtering for smooth trajectory estimation, or learning-based temporal prediction modules that anticipate defect locations based on motion patterns.

Pipeline Architecture: Our framework adopts a sequential three-stage pipeline where information flows unidirectionally from mesh reconstruction to semantic filtering to multi-frame fusion. While this design is computationally efficient and achieves satisfactory performance, it lacks explicit cross-stage feedback mechanisms. For instance, detected defect locations could potentially inform local mesh refinement or accumulated defect confidence could guide adaptive sampling in subsequent frames. Incorporating such iterative refinement loops represents a potential avenue for performance improvement, though at the cost of increased architectural complexity.

Despite these limitations, the current framework demonstrates the feasibility and effectiveness of integrating differentiable geometric reconstruction with vision-based defect detection for road infrastructure monitoring. The experimental results establish that semantic filtering provides substantial reduction in false positives (33.7%), multi-frame fusion significantly boosts detection confidence (2.48 observations per vertex), and the unified BEV representation enables intuitive spatial reasoning for maintenance planning. These contributions provide a solid foundation for future research toward robust, efficient, and deployable road defect mapping systems.

## 5. Conclusions

In this research, we have presented a novel framework for accurate and robust road defect mapping from monocular video sequences by integrating differentiable BEV mesh representation, semantic filtering, and multi-frame temporal fusion. Our differentiable mesh-based BEV representation has enabled efficient scene reconstruction while preserving explicit geometric structure, making it well-suited for infrastructure mapping applications. The semantic filtering strategy effectively eliminated off-road false positives, reducing detection errors by 33.7%, while our multi-frame fusion mechanism with ray-casting projection and EMA-based temporal accumulation successfully aggregated defect observations across frames, enhancing detection confidence while maintaining 3D spatial consistency. Experimental results on real-world driving sequences demonstrated that our framework produced geometrically consistent BEV defect maps with improved detection accuracy compared to single-frame 2D methods, providing valuable spatial information for road maintenance planning and prioritization.

Despite these promising results, several directions remain for future work. The current framework processes frames sequentially, limiting its real-time performance for long video sequences. Investigating efficient parallel processing strategies or incorporating temporal prediction modules could improve computational efficiency. Additionally, developing more robust semantic priors or incorporating uncertainty estimation could enhance generalizability across diverse road types and weather conditions. Extending the framework to incorporate complementary sensing modalities for subsurface defect detection and validating the system on diverse road networks across different geographic regions represent promising directions for practical deployment. In conclusion, this work has demonstrated the feasibility and effectiveness of combining differentiable rendering, BEV representation, and temporal fusion for vision-based road defect mapping, opening new possibilities for automated, scalable, and cost-effective road infrastructure monitoring systems.

## Figures and Tables

**Table 1:** Dataset statistics and scene characteristics.

Scene	Type	Lighting	Traffic	Frames	Vertices	Total Detections
nuScenes-0063	Exit passage	Daytime	Sparse	39	130,770	1710
nuScenes-0064	Parking lot	Daytime	Medium	40	143,857	4908
nuScenes-0200	Parking lot	Daytime	Dense	39	149,635	4233
nuScenes-0283	Right-turn intersection	Daytime	Medium	40	144,924	2160
nuScenes-0655	Complex parking lot	Daytime	Dense	41	143,144	2247
KITTI-00	Urban street	Daytime	Sparse	4541	3,171,274	1452

**Table 2:** Implementation parameters.

Parameter	KITTI	nuScenes
BEV Coverage	600 × 600 m	100 × 100 m
Resolution	0.1 m	0.1 m
Positional Encoding	L = 4	L = 5
Confidence Threshold	0.10	0.05
Road Overlap Threshold	0.5	0.5
EMA Coefficient α	0.3	0.3
Cameras	1 (front)	6 (surround)

**Table 3:** Semantic filtering performance across scenes. The filter successfully removes approximately one-third of non-road false detections, with stable performance across different scenes.

Scene	Total Detections	Filtered	Filter Rate (%)	On-Road Precision (%)
nuScenes-0063	1710	471	27.5	72.5
nuScenes-0064	4908	1656	33.7	66.3
nuScenes-0200	4233	1460	34.5	65.5
nuScenes-0283	2160	732	33.9	66.1
nuScenes-0655	2247	889	39.6	60.4
**nuScenes Average**	**3052**	**1042**	**33.8 ± 4.1**	**66.2**
KITTI-00	1452	536	36.9	63.1

**Table 4:** Multi-frame fusion quality. High observation counts per vertex indicate effective multi-frame aggregation, enhancing defect detection reliability.

Scene	Vertices	Defect Vertices	Coverage (%)	Obs/V	Severity
nuScenes-0063	130,770	130	0.10	2.28	0.132
nuScenes-0064	143,857	375	0.26	2.39	0.153
nuScenes-0200	149,635	329	0.22	2.03	0.127
nuScenes-0283	144,924	166	0.12	2.97	0.184
nuScenes-0655	143,144	162	0.11	2.71	0.191
**nuScenes Average**	**142,466**	**232**	**0.16**	**2.48**	**0.157**
KITTI-00	3,171,274	127	0.00	2.26	0.158

**Table 5:** 3D mapping accuracy. MSR indicates percentage of valid detections successfully projected to mesh. Mapping failures primarily stem from depth estimation limitations.

Scene	Valid Detections	Mapped	MSR (%)	Average Distance (m)
nuScenes-0063	1239	297	24.0	0.18
nuScenes-0064	3252	895	27.5	0.15
nuScenes-0200	2773	667	24.1	0.17
nuScenes-0283	1428	493	34.5	0.14
nuScenes-0655	1358	439	32.3	0.16
**nuScenes Average**	**2010**	**558**	**27.8**	**0.16**
KITTI-00	916	287	31.3	0.12

**Table 6:** Road filtering threshold ablation (Scene-0064). Three threshold settings show highly similar performance, demonstrating method robustness with this parameter.

$\tau_{road}$	Total Detections	Filtered	Filter Rate (%)	Coverage (%)	On-Road Precision (%)
0.3	5052	1715	33.9	0.27	66.1
**0.5**	**4908**	**1656**	**33.7**	**0.26**	**66.3**
0.7	4932	1672	33.9	0.26	66.1

**Table 7:** Confidence threshold ablation (Scene-0064). Lower threshold maximizes recall, with subsequent semantic filtering maintaining precision.

$\tau_{conf}$	Total Detections	Filtered	Valid	Coverage (%)	Defect Vertices
**0.05**	**4908**	**1656**	**3252**	**0.26**	**375**
0.10	4809	1616	3193	0.25	365
0.15	3339	1122	2217	0.18	259
0.20	2523	851	1672	0.14	198
0.25	2064	722	1342	0.11	162

**Table 8:** EMA weight ablation (Scene-0064). Different α values have minimal impact on final performance, consistent with relatively short training cycle.

EMA α	Coverage (%)	Defect Vertices	Total Observations	Obs/V	Severity
**0.5**	**0.26**	**375**	**895**	**2.39**	**0.153**
0.7	0.26	372	893	2.40	0.154
0.9	0.26	372	896	2.41	0.155

**Table 9:** Cross-dataset comparison between nuScenes and KITTI-00.

Metric	nuScenes Average	KITTI-00	Notes
Filter Rate (%)	33.8 ± 4.1	36.9	Stable
Obs/Vertex	2.48	2.26	Consistent
Coverage (%)	0.16	0.00	Dataset-dependent
Average Severity	0.157	0.158	Similar

## Data Availability

The research data used in this study are publicly available autonomous driving datasets. The KITTI dataset can be accessed at http://www.cvlibs.net/datasets/kitti/ (accessed on 15 December 2024) and the nuScenes dataset at https://www.nuscenes.org/ (accessed on 15 December 2024). Both datasets require registration and acceptance of their respective terms of use.
